# The Micromorphological Research of the Internal Structure of Chairside CAD/CAM Materials by the Method of Scanning Impulse Acoustic Microscopy (SIAM)

**DOI:** 10.2174/1874210601812010125

**Published:** 2018-01-31

**Authors:** Kristina E. Goryainova, Egor S. Morokov, Marina V. Retinskaja, Fedor S. Rusanov, Samvel V. Apresyan, Igor Yu. Lebedenko

**Affiliations:** 1 The Department of Prosthetic Dentistry of Medical Faculty, The Peoples' Friendship University of Russia, 117198, Moscow, Russia; 2 The N.M. Emanuel Institute of Biochemical Physics RAS, IBCP RAS, 119334, Moscow, Russia; 3 Federal State Institution, Central Research Institute of Dental and Maxillofacial Surgery, Ministry of Health, Russian Federation, 119034, Moscow, Russia; 4 Scientific and Technological Center of Unique Instrumentation RAS, IBCP RAS, 117342, Moscow, Russia

**Keywords:** CAD/CAM, CEREC, Chairside, Hybrid ceramic, Leucite glass-ceramic, Feldspatic ceramics, PMMA, Internal structure, Microstructure, Porosity, Acoustic microscopy

## Abstract

**Aim:**

The aim of the present work was to compare the elastic properties and internal structure of 4 different CAD/CAM chairside materials, by the method of Scanning Impulse Acoustic Microscopy (SIAM).

**Methods:**

Four chairside CAD/CAM materials with different structures from hybrid ceramic (VITA Enamic, VITA Zahnfabrik), feldspatic ceramic (VITABlocs Mark II, VITA Zahnfabrik), leucite glass-ceramic (IPS Empress CAD, Ivoclar Vivadent) and PMMA (Telio CAD, Ivoclar Vivadent) were examined by Scanning Impulse Acoustic Microscope (SIAM).

**Results:**

The results of micromorphological research of CAD/CAM chairside materials using SIAM method showed differences between the internal structures of these materials. The internal structure of feldspatic and glass-ceramic samples revealed the presence of pores with different sizes, from 10 to 100 microns; the structure of polymer materials rendered some isolated defects, while in the structure of hybrid material, defects were not found.

**Conclusion:**

Based on the results obtained from the present study, in cases of chairside production of dental crowns, it would be advisable to give preference to the blocks of hybrid ceramics. Such ceramics devoid of quite large porosity, glazing for CAD/CAM crowns made from leucite glass-ceramic and feldspatic ceramic may be an option. For these purposes, commercially available special muffle furnace for clinical and laboratory individualization and glazing of ceramic prostheses were provided. Further studies are needed to confirm the evidence emerging from the present research.

## 1 INTRODUCTION

Over the past decades, CAD/CAM technology has achieved the best optimization of the process, obtained blocks made from different materials complying with aesthetic requirements, as well as biological principles. High strength characteristics of CAD/CAM chairside materials are associated primarily with the peculiarities of their microstructure. Modern materials are based on ceramics as structure with nanoscale crystalline grains and intergranular minimum intervals.

Characteristics of structure, grain size and their packing density in large part set mechanical and strength properties of the materials. Modern dentists make a choice of material for CAD/CAM chairside restorations based on the knowledge gained from official information leaflets [1]. Rather frequent is such situation when the indications of different (chemical composition and structure) materials are exactly the same (inlays, onlays, overlays, crowns, veneers).

In such cases, the choice of material for clinical application is difficult without considering the peculiarities of physical-mechanical properties and structure of blocks for the CAD/CAM technology [2].

The aim of the present work was to compare the elastic properties and internal structure of 4 different CAD/CAM chairside materials, by the method of Scanning Impulse Acoustic Microscopy (SIAM).

## 2 MATERIALS AND METHODS

Studies were conducted on samples made from various types of materials for CAD/CAM chairside techniques:

- Hybrid ceramic VITA Enamic (VITA Zahnfabrik, Germany);
- Feldspatic ceramic VITABlocks Mark II (VITA Zahnfabrik, Germany);
- Leucite glass-ceramic IPS Empress CAD (Ivoclar Vivadent, Liechtenstein);
- PMMA material Telio CAD (Ivoclar Vivadent, Liechtenstein).

Available information about the percentage of oxide compounds in blocks of feldspatic ceramics were: SiO_2_ - 56-64%, Al_2_O_3_ - 20-23%, Na_2_O - 6-9%, K_2_O - 6-8%, CaO - 0,3-0, 6%, TiO_2_ - 0,0-0,1%. Leucite glass-ceramic blocks consist of SiO_2_, Al_2_O_3_, K_2_O, Na_2_O, CaO and other oxides and pigments. Blocks of hybrid ceramics have 86% of feldspatic ceramics and 14% of polymer component. The structure of this material is an interpenetrating ceramic and polymer matrix. The ceramic structure hybrid ceramics is: SiO_2_ - 58-63%, Al_2_O_3_ - 20-23%, Na_2_O_6_ -11%, K_2_O_4_ - 6%, B_2_O_3_ - 0.5-2%, CaO - <1%, TiO_2_ - <1% and others. Polymer blocks contain 98% of polymethylmethacrylate and approximately 1% of pigments.

Samples of materials were made in the form of plates (size 15х5х3 mm) with plane-parallel sides. The samples were alternately fixed on the object table placed in the cup with distilled water (27 °C) and were studied using the acoustic microscope SIAM-2, developed at the Institute of biochemical physics at Moscow (Russia) [3, 4]. When probing for sample, ultrasonic pulses pass through the immersion liquid, reflect from the front surface of the sample, the elements of the internal structure and the bottom of the sample.

The reflected signal consists of signals separated by intervals which are determined by the propagation time of the probing beam from one obstacle to another. These time differences allow to select the signals received at different depths in the sample volume and build the bitmap of one-dimensional (B-scans) and two-dimensional (C-scans) acoustic images of the internal microstructure.

**Fig. (1):**
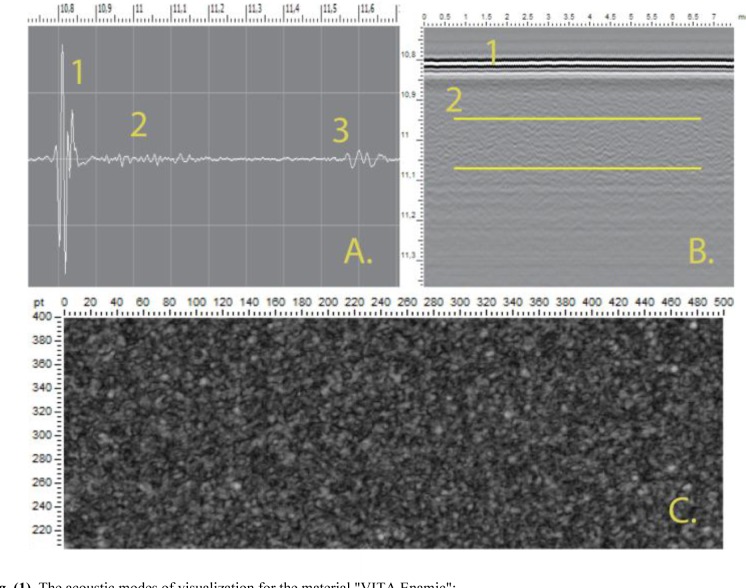
The acoustic modes of visualization for the material "VITA Enamic": **a**) Oscillogram (A-scan) **1** - the signal reflected from the sample surface, **2** - the signals from the elements of the internal structure, **3** - the signal from the lower limit of the sample; **b**) Vertical cut of the sample (B-scan); **1** - the surface of the sample; **2** - focus of acoustic lens; **c**) Two-dimensional acoustic image (C-scan), the structure of the image corresponds to a layer in the sample, its position and thickness are shown in A and B-scans.

Studies on samples were performed with acoustic lens having a working frequency of 100 MHz and an aperture of 11°. We analyzed oscillograms (A-scans), images of the internal structure of samples obtained in the form of transverse vertical slices (B-scans) and the images of the internal structure with a predetermined thickness at a predetermined depth in the sample (C-scans) (Fig. **1**).

Time delay between pulses reflected from the top and bottom surfaces were defined by the local values of velocity of longitudinal and transverse waves. On the oscillograms by the time delay, the signal was determined reflected from the lower boundary of the sample and formed during the passage of a longitudinal wave through the volume of material; and signal generated by the conversion of the sound modes when passing through the volume of material.

The resulting time of propagation of the longitudinal and transverse waves in conjunction with the known thickness of the material allowed us to determine the velocity of longitudinal C_L_ and transverse C_T_ sound waves in the material. The density ρ of the materials was obtained by the method of hydrostatic weighing by the balance KERN Alt 220.

Based on the data of density of the studied materials, the local values of bulk (K) and shear (G) modules of elasticity of the materials were calculated as follows:

(1) $$G=C_{T}^{2}.p$$

(2) $$K=C_{L}^{2}.p-\frac{4}{3}.C_{T}^{2}.p$$

- C_L_ - the longitudinal wave velocity, km/s,

- C_T_ - the transversal wave velocity, km/s,

- ρ - density of the material, g/cm3.

Also we calculated the Young's module (E) and Poisson's ratio (µ) [4]:

(3) $$E=\frac{9.K.G}{3.K+G}$$

(4) $$\mu =\frac{E}{2G}-1$$

- K - the bulk module of elasticity, GPa

- G - the shear module of elasticity, GPa.

## 3 RESULTS

On the oscillograms (A-scans), time delays of signals due to the passage of longitudinal waves and conversion of longitudinal and transverse waves were determined. Results of the calculated speed of propagation of longitudinal and transverse ultrasonic waves inside materials are shown in Tables **1**, **2** present the results of calculating the density of materials. Table **3** presents the results of the calculation of the elastic modules: bulk module, shear module, Young's module and Poisson's ratio. Ceramic materials characterized by high, (in comparison with other research materials) values of shear modules and modules of compression, have high stiffness (E = 70 GPa) and low plastic properties (µ = 0,19), which determine the propensity of this type of material to brittle fracture.

The polymer group of materials has the lowest values of modules of elasticity, so respectively is subjected to deformation and fracture under low loads.

Plastic properties of hybrid ceramic (due to its dual internal structure) are best compared to leucite glass ceramic properties, but the strength characteristics of hybrid ceramic are lower in 1.5 times.

The obtained results must be considered in the clinical dental practice in the choice of materials for the manufacturing of dental crowns with different wall thicknesses. When a thin-walled permanent dentures, ceramic and hybrid CAD/CAM blocks should preferably be used. It is possible that high values of deformation of polymeric crowns under the functional loads due to the low elastic modulus of these materials cause partial and full recementation and “edge permeability” at the borders of hard tissue of teeth and thin walls of polymer dentures.

Analysis of the internal microstructure of the samples was performed under the same preset scan settings (amplification of the ultrasonic signal, the scan step *etc*). Bitmap construction of acoustic images showed the brightness point in the image depending on the amplitude of the reflected signal at the same point. Fig. (**2**) shows acoustic images of the internal structure of the samples (scans). The structure of the samples displays a depth of 0.8 mm from the surface and the thickness of the layer images - 50 µm. Bright dots in the images correspond to the pores in the volume of the samples. Most of them were observed in the volume of feldspatic ceramic and glass ceramic materials, the size of which varies from a few microns to hundred of microns.

**Fig. (3):**
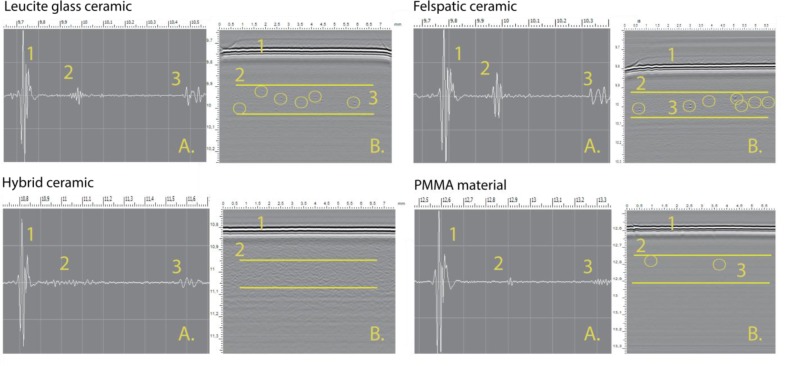
The acoustic modes of 4 different CAD/CAM chairside materials: **A**) Oscillogram (A-scan) **1** - the signal reflected from the sample surface, **2** - the signals from the elements of the internal structure, **3** - the signal from the lower limit of the sample; **B**) Vertical cut of the sample (B-scan); **1** - the surface of the sample; **2** - focus of acoustic lens; **3** - The pores in the internal structure of the material

In the volume of the hybrid ceramic uniform, two-phase structure (ceramic and polymer) was observed. Variation of dark and gray areas in the image corresponds to the amplitudes of the reflection on the border of two phases in the material volume. The volume of the polymeric material demonstrates a homogeneous structure (dark background) with a low content of pores and air inclusions (bright spots on image). Thus, we found that the volume of blocks (obtained by pressing) from ceramic materials had pores with the size significantly exceeding the dimensions of the crystalline grains and the spaces between them, exceeding the common size of dentures precision (50 microns) (Fig. **3**). Such defects can create points of retention of dyes and microorganisms, and therefore, influence the longevity of restoration.

## 4 DISCUSSION

In recent years, CAD/CAM technology reached the greatest development with the method called “chairside”. The advantages of this method/technique over the labside technique are: time saving process, patient's involvement in the process of making restoration and the need of a dental laboratory [3-5]. Often different dental materials have the same indications for use. Despite the relevance of this topic, there is lack of necessary information in academic literature about the peculiarities of application of different chairside materials in dentistry. We conducted a study on the scientific literature contributed by domestic and foreign authors and identified the fragmented nature of research on this topic. To study one of the most important parameters of the dental CAD/CAM materials (microstructure), we decided to use а scanning impulse acoustic microscopy. With the method of Scanning Impulse Acoustic Microscopy (SIAM), we studied 3 groups of CAD/CAM chairside materials and detected differences in the structure of blocs of ceramic, hybrid ceramic and polymeric materials for CAD/CAM technology [6-10].

We obtained acoustic images of internal structures of CAD/CAM chairside materials which were studied for their elastic properties. We proved that the materials used for the solution of similar clinical tasks are fundamentally different in their elastic characteristics: from soft (flexible) polymeric materials to hard (prone to brittle fracture) ceramic materials [11, 12]. Study of the internal microstructure of the CAD/CAM chairside materials showed the presence of fine-pored structure inside leucite glass ceramic samples and feldspatic ceramic samples. The size of the largest of them was measured using software of the microscope. The maximum detected pore size in leucite glass ceramic samples was 70±15 µm. The maximum detected pore size in feldspatic ceramic samples was 100±15 µm [6, 12-16]. Therefore, the size of pores in ceramic ranged from 10 to 100 microns. In the volume of polymer materials, isolated point defects of internal structure were found. In the structure of hybrid material, defects were not detected. Our results indicate the possibility of the presence of relatively large pores (up to 100 microns) as surface defects on ceramic crowns made by CAD/CAM chairside technology. CAD/CAM chairside technology requires full refuse of dental laboratory during the process of dental treatment; the final step of producing crown is only polishing [6, 12-16]. But mechanical polishing cannot polish out surface defects (from 10 to 100 microns) without a significant change in the shape and the size of the crown.

## CONCLUSION

Apparently, in cases of chairside production of dental crowns, it is necessary to give preference to the blocks of hybrid ceramics. Such ceramics devoid of quite large porosity, glazing for CAD/CAM crowns made from leucite glass-ceramic and feldspatic ceramic may be an option. For these purposes, commercially available special muffle furnace for clinical and laboratory individualization and glazing of ceramic prostheses were provided. Such equipment even more increases the cost of manufacturing of CAD/CAM chairside ceramic crowns compared to using blocks of hybrid ceramics for the same goals [12-16].

## Figures and Tables

**Fig. (2):**
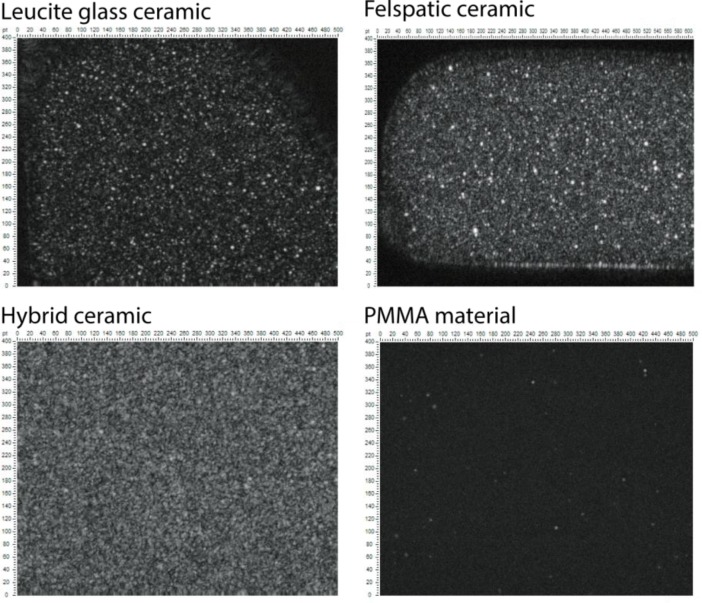
Acoustic images (C-scans) of bulk microstructure of the dental samples. Operating frequency of the lens 100 MHz. The scan field - 6x7,5 mm^2^

**Table 1:** The speed of propagation of longitudinal (C_L_) and shear (C_T_) waves in the samples of dental materials.

**Material**	**Samples**	**C_L_, km/sec**	**C_T_, km/sec**
Hybrid ceramics	VITA Enamic	4,62	2,81
Ceramics	IPS Empress CAD	5,46	3,37
	VITABlocs Mark II	5,72	3,53
Polymers	Temp Premium	2,22	1,03
	Telio CAD	2,71	1,41

**Table 2:** The density (*ρ)* of dental materials.

**Material**	**Samples**	***ρ*, g/cm^3^**
Hybrid ceramics	VITA Enamic	2,148±0,06
Ceramics	IPS Empress CAD	2,451±0,07
	VITABlocs Mark II	2,452±0,07
Polymers	Temp Premium	1,195±0,04
	Telio CAD	1,179±0,03

**Table 3:** The value of bulk (K) and shear (G) modules of elasticity of the material, Young's modulus (E) and Poisson's ratio (µ).

**Material**	**Samples**	**G, HPa**	**K, HPa**	**E, HPa**	**µ**
Hybrid ceramics	VITA Enamic	16,93	23,24	40,86	0,21
Ceramics	IPS Empress CAD	27,88	36,06	66,50	0,19
	VITABlocs Mark II	30,48	39,73	72,82	0,19
Polymers	Temp Premium	1,26	4,23	3,43	0,36
	Telio CAD	2,33	5,55	6,13	0,32
